# Motivation to exert effort dynamically adapts to reward context and environmental controllability

**DOI:** 10.3758/s13415-026-01484-4

**Published:** 2026-08-31

**Authors:** Bingjie Li, Tim Vriens, Yibei Liu, Mingqian Guo, Karin Roelofs, Eliana Vassena

**Affiliations:** 1 Behavioural Science Institute, Radboud University, Thomas van Aquinostraat 4, 6525 Nijmegen, GD Netherlands; 2 Donders Center for Cognitive Neuroimaging, Radboud University, Nijmegen, Netherlands; 3 Institute of Cognitive Sciences and Technologies, CNR, Rome, Italy

**Keywords:** Motivation, Effort-based decision-making, Environmental controllability, Reward context, Depression

## Abstract

Working hard to pursue our dreams should get us there sooner or later. However, no matter how hard we try, sometimes, things are simply beyond our control, and our efforts may even be detrimental. Swiftly adapting our efforts to the controllability of the environment is a critical skill that is impaired in many neuropsychiatric disorders. Yet, how exactly we calibrate our efforts to the controllability and the richness of the environment (i.e., the overall amount of reward available in the current context) remains largely unexplored. We developed the novel Controllability and Mental Effort Task (CoMET) to investigate how varying controllability and reward context impact the motivation to exert effort for reward (*n* = 61). Importantly, we tested the impact of controllability while controlling for average reward rate. As hypothesized, participants were more willing to exert effort in controllable environments than in uncontrollable ones and adjusted their decisions to exert effort dynamically as a function of varying controllability. Their willingness to exert effort was also sensitive to the reward context: in reward-poor contexts, participants engaged in more effort when the momentary reward was high. Drift Diffusion modeling of decisions to exert effort showed that controllability, high momentary reward, and low effort enhanced the value integration process with increased drift rates, and controllability specifically increased decision threshold. Interestingly, individuals with higher subclinical depression traits were more sensitive to controllability, showing diminished motivation, particularly in an uncontrollable high-reward context. Taken together, these results indicate that people dynamically track multiple environmental features and integrate them into their effort allocation strategies.

**Supplementary Information:**

The online version contains supplementary material available at https://doi.org/10.3758/s13415-026-01484-4.

“We cannot choose our external circumstances, but we can always choose how we respond to them.” –Epictetus

When facing constant challenges, each person has boulders to bear—or even push uphill—in their own life. In such straining circumstances, the Greek Stoic philosophers emphasized the distinction between what lies within our control and what does not. Calibrating our efforts wisely, by taking into account the level of control we hold over our environment, is a critical skill. Motivation to exert effort to achieve desired outcomes has been extensively studied in many disciplines. In psychology and neuroscience, motivation has been conceptualized as a tradeoff between the value of desired outcomes and the cost required to obtain them (Brehm & Self, 1989; Silvetti et al., 2018; Treadway et al., 2012). One ubiquitous example of such costs is the mental or physical effort required to achieve a goal (Kool et al., 2017; Vassena et al., 2019a). Individuals avoid exerting effort if possible (Kool et al., 2010) and perceive effort as aversive and frustrating (David et al., 2024). The value of a desired outcome is discounted, i.e., reduced proportionally to the amount of effort required (Apps et al., 2015). The willingness to invest effort has been investigated across a variety of cognitive tasks (Apps et al., 2015; Constantino & Daw, 2015; Suzuki et al., 2020), including working memory, response inhibition, and task switching. In sum, to maximize reward and minimize effort cost, we decide to exert effort when it is worth it (David et al., 2024; Silvestrini et al., 2023).

However, exerting effort does not always lead to the desired outcome. At times, the situation is simply beyond our control, regardless of how much effort we exert. Early studies on the impact of controllability have investigated the phenomenon of “learned helplessness” (Seligman & Maier, 1967). Animals exposed to uncontrollable stressors in the form of inescapable shocks showed reduced motivation to avoid aversive outcomes even later on, where controllability was restored (Seligman, 1972). This maladaptive generalization has been considered an important mechanism in the development of psychopathology (Huys & Dayan, 2009; Maier & Seligman, 2016; Tiwari et al., 2023).

Recently, controllability has been operationalized as the extent to which one’s actions can influence the current environment, proposing that the subjective estimation of controllability is crucial in adjusting one’s actions (Moscarello & Hartley, 2017). This adjustment entails arbitrating between proactive, goal-directed behavioral responses (adaptive in a controllable environment) and reactive, passive responses (adaptive in an uncontrollable environment). Balancing these control modes according to the situation is essential, and this balance should depend on the estimated controllability (Dorfman & Gershman, 2019; Ligneul et al., 2022).

To quantify the balance between control modes, Ligneul et al. (2022) adopted an *information-theoretic approach,* operationalizing controllability as the extent to which one’s actions drive the environment to transition from its current state to a future state (in the absence of reward). A complementary perspective considered the *efficacy of control allocation* (Grahek et al., 2019; Shenhav et al., 2013, 2021), which is defined as the extent to which exerting control leads to the expected outcome (i.e., efficacy) when performing cognitively effortful tasks. Higher expected efficacy is associated with faster and more accurate task performance (Frömer et al., 2021). This perspective specifically focused on control adjustments during effortful task performance (without considering decision-making) with the merit of integrating the controllability and effortful performance angles. Whether varying controllability influences choices to engage in effort *during decision-making* remains to be clarified.

Notably, the motivation to exert effort does not only depend on controllability but also on the reward context (intended as the amount of reward available in the environment) (Ly et al., 2019). Typically, people track momentary changes in reward but also the average reward rate in the environment and adapt their effort allocation accordingly (Botvinick & Braver, 2015; Wittmann et al., 2020). The same reward amount can lead to higher effort allocation in a reward-poor context compared with a reward-rich context (Otto & Vassena, 2021), indicating that the reward value is scaled to the reward context (Otto & Daw, 2019; Seymour & McClure, 2008). We perceive the same reward very differently depending on whether we find ourselves in a reward-rich or reward-poor environment (Palminteri & Lebreton, 2021), which affects choices (Rangel & Clithero, 2012) and effort allocation (Otto & Vassena, 2021).

Higher reward availability has been linked to increased response vigor (Niv et al., 2007), because it intrinsically carries a higher opportunity cost of time: if rewards are abundant, responding more slowly entails forgoing additional rewards (Wang et al., 2019). An abundance of rewards sometimes lead to disengagement of cognitive resources (Boureau & Dayan, 2011; Scholz et al., 2022), because one can achieve sufficient rewards even with lower effort. This is in line with evidence showing faster responses but more errors in higher reward context in cognitive control tasks (Otto & Daw, 2019). To sum up, converging evidence indicates that the impact of momentary rewards is relative to the reward context, and reward-rich contexts also promote action vigor while inhibiting instrumental effort allocation. The interplay of these multifaceted context effects with environmental controllability remains to be clarified.

While behavioural performance measures have been employed to quantify the impact of controllability and reward context on control allocation, the underlying cognitive mechanisms remain debated. The Drift Diffusion Modeling (DDM) framework (Ratcliff & McKoon, 2008) has proven beneficial in dissociating two forms of motivated control adjustments: attention allocation and value integration (reflected in the drift rate parameter) and decision caution (reflected in the threshold parameter) (Leng et al., 2021; Yee et al., 2024; Van Veen et al., 2008; Erhan & Balcı, 2017). Expecting higher rewards is associated with increased attention allocation and reduced caution (Leng et al., 2021), and higher performance efficacy is linked to increased attention allocation as well (Grahek et al., 2023). While these studies mainly focused on *motivated performance*, during value-based decision-making, the DDM accumulation process is thought to capture *value integration* (Milosavljevic et al., 2010). Hence, this approach could help to elucidate whether changes in value difference processing, decision caution, or both may drive the impact of controllability and reward context on effort allocation decisions.

It is important to understand better how controllability and reward context influence motivation to exert effort (and the underlying cognitive mechanisms), because motivational alterations are a critical symptom in stress-related disorders (Hermans et al., 2025), notably associated with increased susceptibility to stressors, including exposure to low controllability or reward-poor environments (Mehta et al., 2023). Low perceived control is associated with increased severity of depression (Dulin et al., 2013) and anxiety symptoms (Gallagher et al., 2014). Depression and anxiety have been consistently linked with altered effort exertion (Berchio et al., 2019; Treadway et al., 2009). Elucidating the interplay between controllability and reward context in modulating effort allocation may bring a better understanding of how vulnerability to motivational deficits arises in psychopathology, particularly in the presence of uncontrollable stressors (Baratta et al., 2023).

In sum, how controllability and reward context are swiftly integrated to guide adaptive decisions remains unclear. In this study, we propose a novel approach integrating the information-theoretic and control efficacy perspectives. We reframe the estimation of controllability as a *reinforcement meta-learning* problem (Silvestrini et al., 2023; Silvetti et al., 2018) and propose that people dynamically integrate all decision-relevant variables, including required effort, probability of success, reward context, and environmental volatility. The integration of these variables leads to the decision to invest effort or to disengage when exerting effort is no longer valuable. We define an environment controllable when successful effort exertion leads to the desired outcomes (in line with prior formalizations of controllability, Huys & Dayan, 2009). With a novel paradigm, we investigate whether and how individuals dynamically adjust their willingness to exert effort and their effortful performance to varying controllability and reward context. We test whether momentary rewards are valued differently depending on the context in which they are presented and whether the reward context modulates the impact of controllability. Finally, we test whether higher psychopathology traits (depression and anxiety) make people more sensitive and potentially more vulnerable to lower controllability.

First, we hypothesize that people dynamically track controllability by reducing their willingness to exert effort in uncontrollable blocks and that momentary reward value will be scaled relatively to the average reward context (Otto & Vassena, 2021). This would suggest that controllability and reward context are tracked and integrated to inform decisions. Second, we investigate the putative underlying mechanisms by applying computational modeling with a Drift Diffusion Model. We test whether behavioral differences are primarily driven by value integration (drift rate) or by caution (decision threshold). Finally, we hypothesize that sub-clinical psychopathological traits (depression and anxiety levels) are associated with increased susceptibility to uncontrollability (Baratta et al., 2023).

## Methods

This study was preregistered on the Open Science Framework before data analysis (https://doi.org/10.17605/OSF.IO/A6Q2E).

### Participants

We recruited 70 healthy students from Radboud University by using the Sona Participant Recruitment online system. The exclusion criteria are customary for this task (Vassena et al., 2019a) and were also pre-registered. Two participants were excluded due to incomplete data, and one was excluded due to technical issues during data collection. One participant was excluded, because they had more than 50% missing trials and a corresponding accuracy rate lower than the chance level (33%) in the second part of the study. Five participants were excluded, because they showed insufficient variability in their decision-making behaviour (i.e., an average acceptance rate higher than 95%). A total of 61 participants (47 females, 12 males, and 2 who preferred not to disclose their gender) were included in the behavioral analysis. Based on previous studies, a sample size of 62 participants (with a small effect size, F = 0.15, power = 0.80, and alpha = 0.05) was estimated. Inclusion criteria were 1) bachelor students eligible to earn credits for participation in the study; 2) age between 18 and 40 years; 3) no current diagnosis or history of neurological or psychiatric disorders; 4) not color-blind. Written consent was obtained from all participants before they participated in the study. As compensation, participants received course credits (1 credit per hour, ranging from 1 to 1.25, depending on the total duration), with the time variation attributed to the RT calibration. Participants who performed among the top 50% had the chance to win a 5-euro digital Bol.com voucher. This study was conducted under the light track ethical approval procedure by the Ethics Committee for Social Science (ECSS) of Radboud University (#ECSW-LT-2023-12-5–89357) and complies with the European General Data Protection Regulation (GDPR).

### Procedure

The experiment lasted approximately 60 min, with a maximum duration of 70 min, and the duration varied due to the calibrated personalized reaction time (RT). After providing informed consent, participants performed the Controllability of Mental Effort Task (CoMET). After completing the tasks, participants were asked to complete subjective ratings on a 7-point Likert scale regarding their motivation by the momentary reward, the perceived difficulty of the effortful calculations, and whether they thought the chance of obtaining rewards changed throughout the task. The exact questions were: “How motivating did you find these reward points: 30/40/50 points?” (On a 7-point scale, ranging from 1 [“not at all motivating”] to 7 [“very motivating”]); “How difficult did you find the calculation proceeded by this picture?” upon viewing each of the four condition cues (On a 7-point scale, ranging from 1 [“not at all difficult”] to 7 [“very difficult”]); “How much did you feel your chances of getting reward points by calculations changed following each diamond picture? Please rate, on a scale from 1 to 7.” (On a 7-point scale, ranging from 1 [“no change”] to 7 [“a significant change”]).

After completing the subjective ratings, participants filled in four questionnaires: the Depression Anxiety Stress Scales (DASS-21) (Lovibond & Lovibond, 1995); the Perceived Stress Scale (PSS-10) (Cohen et al., 1983); the Daily Hassles (general stressors) (Veer et al., 2020); and the Learned Helplessness Scale (Quinless & Nelson, 1988). The Depression and Anxiety subscale scores were the primary variables of analysis. The Daily Hassles, the Perceived Stress Scale, and the Learned Helplessness Scale were administered for exploratory purposes, outside the scope of the current manuscript, to compare with a previous study.

After collecting data for the entire sample, 50% of the top performers entered a lottery to win a € 5 digital voucher. Vouchers were awarded by email.

### Controllability of mental effort task (CoMET)

We developed the novel CoMET based on an established effort-based decision-making and performance task (Vassena et al., 2014, 2015, 2019a). CoMET encompasses calibration and main task phases, including decision-making and performance*.*

### Calibration phase

The calibration procedure was developed by Vriens & colleagues (2026). Prior to the main task phase, RT was calibrated based on individual calculation performance. This procedure aimed to control the task difficulty for each participant by optimizing a personalized RT based on the RT of the correct trials and the number of incorrect trials. The personal calibrated RT (*M* = 1608.56 ms, *SD* = 100.47 ms) was used for the following *main task phase* to achieve an average accuracy rate of approximately 80%. To this end, we included a short pre-calibration block (20 trials), a calibration block (16 trials), and a post-calibration block (20 trials). We asked participants to perform a series of calculations of varying difficulty levels. During the pre-calibration block, participants were asked to respond within 3000 ms, and they received performance feedback (i.e., correct, incorrect, or too late). During the calibration block, the personalized RT was calibrated and updated trial by trial based on participants’ performance in this block. Participants were given 3000 ms to answer the calculations, with response feedback indicating whether the answer was correct, correct but too late, incorrect, or too late. The feedback “correct but too late” was given when the answer was correct, but the RT was longer than the calibrated RT. The calibrated RT was calculated to achieve an average accuracy rate of 75%. We expected the overall accuracy on the main task to increase slightly due to practice effects during the experiment. During the post-calibration block, participants were asked to respond within the calibrated RT, with feedback provided (correct, incorrect, or too late).

### Main task phase: Decision-making and performance

The main task started with a short practice session (10 trials)*.* The task consisted of eight blocks, each comprising 40 regular trials and eight bonus trials. On each trial, participants were asked to decide whether to engage in cognitive effort (i.e., performing arithmetic calculations of varying difficulty, easy or hard, easy: e.g., 4 + 1 + 1; hard: e.g., 4 + 7 + 9) for potential rewards (i.e., 50 or 40 points). They were instructed to decide whether to engage based on whether the outcome was worth the effort, and the more points they made, the higher their chance of winning an extra monetary voucher.

**Fig. 1:**
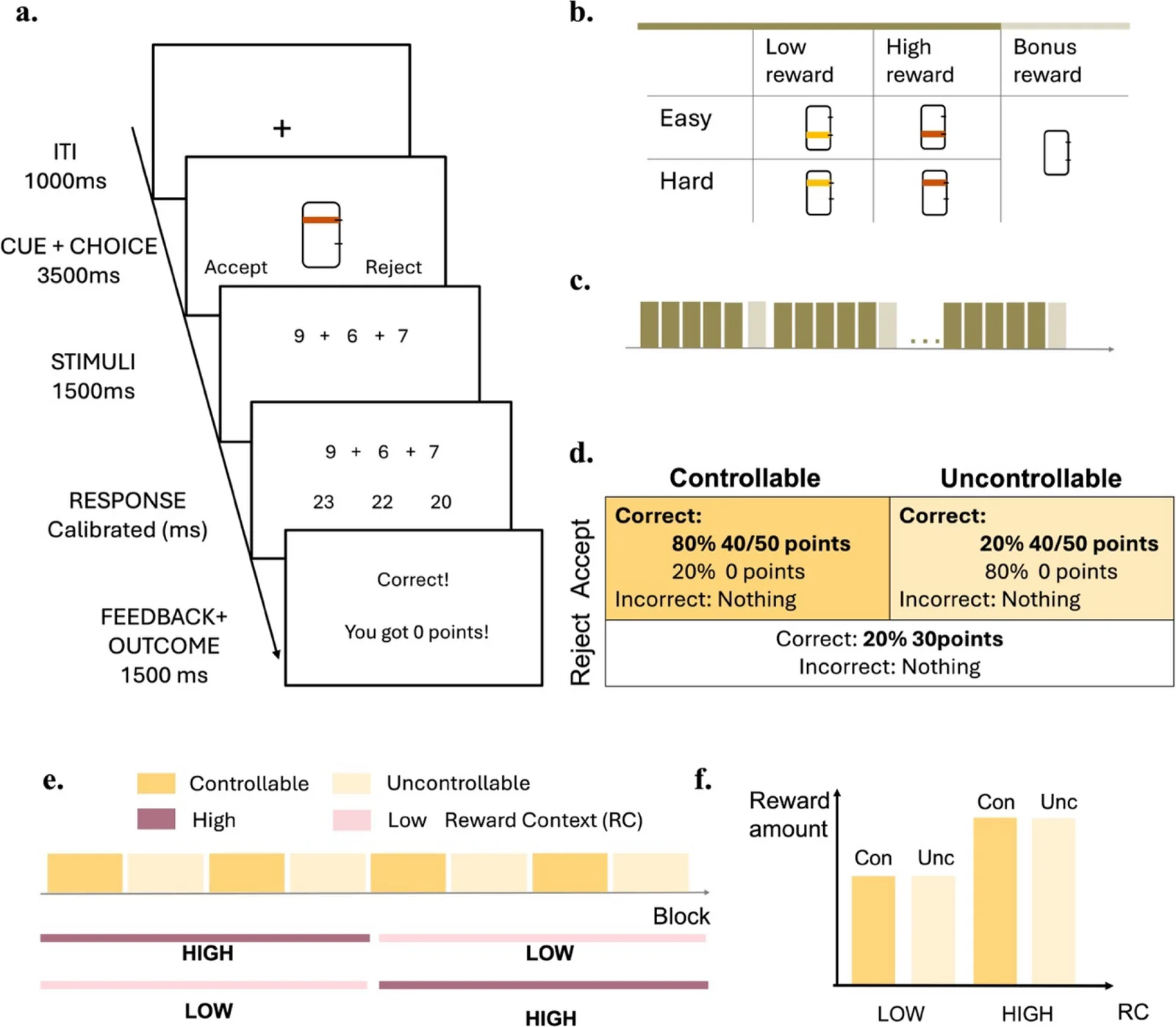
CoMET design. *Note.* **a** Trial Structure. Every trial consisted of an intertrial interval (1000 ms), a cue and choice phase (3500 ms), a calculation stimulus phase, a response phase (calibrated RT), feedback and outcome phase (1500 ms) with feedback (correct, incorrect, or too late) was shown (500 ms), followed by a reward outcome (1000 ms). Each bonus reward trial consisted of an intertrial interval (1000 ms), a cue and choice phase (3500 ms), and an outcome phase (1000 ms). **b** Motivational cues. The cues appeared at the beginning of each trial: A color-filled thermometer marked the start of a regular trial. The empty thermometer marked the start of a reward bonus trial. The position of the horizontal bar in the thermometer indicated the difficulty of the upcoming calculation (i.e., lower: easy, higher: hard). The color of the bar indicated whether the upcoming potential reward was low or high (i.e., light orange: low reward (40 points), dark orange: high reward (50 points). **c** Trial structure within blocks. The time course within a block is divided into mini blocks. Each mini block consisted of five typical trials and one bonus reward trial. The task consisted of 8 blocks, each comprising 40 trials and eight bonus trials, for a total of 384 trials. Bonus Reward Trial: Participants do not need to perform any calculations and will receive points. They are instructed that these points are not dependent on their performance. **d** Successful effort exertion and outcome contingencies in controllable and uncontrollable blocks. **e** Block order. Sequence of the blocks, including controllability and reward context. **f** Average reward amount across blocks

At the beginning of each trial, a cue was displayed, showing a thermometer (Fig. 1). The cue indicated the difficulty and potential reward associated with the upcoming calculation. Upon seeing the thermometer, they could decide whether to accept or reject the given difficulty level calculation for the given reward amount. If they reject, they will perform a very easy calculation (e.g., 6 + 0 + 0) to win the smallest reward (i.e., 30 points). The position of “accept” or “reject” on the screen was randomized. Participants were instructed to press either the left or right key to select the corresponding option on the left or right. Following their decision, the calculation was displayed. Then, participants mentally calculated and selected one of three possible answers. Participants were instructed to respond by pressing the left, middle, or right key to select the corresponding answer given its position. The RT limit was individually calibrated based on the calibration phase. Subsequently, feedback and outcomes were shown. Feedback was aligned with the response, indicating whether the answer was correct, incorrect, or submitted too late (if the response was not received within the specified time). Every five regular trials, one bonus reward trial was presented. At the beginning of the trial, an empty black thermometer was displayed, and participants were instructed to do nothing and that they would receive a random amount of reward points (bonus reward trials) unrelated to their performance.

Controllability was manipulated by varying the likelihood that successful effort exertion led to the desired outcome. Of the eight task blocks, four were controllable and were preceded by a horizontal diamond picture. The other four were uncontrollable and were preceded by a vertical diamond picture. Participants were informed that the diamond picture indicated that something might change but were not instructed on the specific meaning of the picture. Controllable blocks yielded an 80% probability of gaining points (40 or 50 points) when participants engaged in the task and upon successful effort exertion (i.e., provided a correct answer). Conversely, uncontrollable blocks resulted in a 20% chance of reward upon successful effort exertion. In all blocks, rejection to engage in the task with the correct answer on the simple calculation resulted in a 20% chance of the smallest reward (30 points). All incorrect answers resulted in zero points. The task alternated controllable and uncontrollable blocks, starting with a controllable block. Importantly, the average reward rate was controlled across controllable and uncontrollable blocks. This was achieved by delivering bonus points after every five regular trials in the bonus trials. The bonus reward was adjusted based on the amount of performance-dependent reward accumulated in the preceding five trials and a predetermined average reward rate. Participants were informed that these bonus points were not dependent on their performance.

To investigate the impact of reward context, the average reward rate was varied across two contexts, each comprising four blocks: a high-reward context and a low-reward context. The order of the two reward contexts was counterbalanced across participants: half of the participants experienced four high-reward context blocks, followed by four low-reward context blocks, and the other half experienced the opposite order. The average reward rates in the high-reward context were approximately 1.35 times (ranging from 1.30 to 1.40) higher compared with those in the low-reward context. This manipulation allowed for the dissociation of the impact of average reward rate and controllability on willingness to exert effort.

### (Generalized) Linear mixed model (GLMM)

We analyzed the behavioral data using mixed-effects models. Our primary interest is the proportion of accepted trials (i.e., the acceptance rate) during the decision-making phase, which indexes the willingness to exert effort. We fitted a logistic regression model to the acceptance rate, using the following variables as regressors: effort level, momentary reward level, controllability, reward context, and all possible interactions between these variables. To reduce the type I error, we used the so-called maximal design in which all regressors were treated as both fixed and random effects (Barr et al., 2013). Note that these variables are all categorical variables (i.e., effort, reward level, controllability, and reward context). We used sum-to-zero coding (coded as −0.5 and 0.5) for these variables. The mixed-effects logistic regression model was performed in R (v4.3.1; R Core Team 2023) using the package lmer4 (v2.0–1) (Bates et al., 2015). Detailed implementation and significance tests of the mixed effect model follow the recommendations from the Standard Operating Procedures for Using Mixed-Effects Models (Figner et al., 2024). We obtained a Type-III *p*-value using the Likelihood Ratio Test with the Anova () function in the car package (Fox & Weisberg, 2019) and reported the χ^2^ and *p*-value. Fixed-effect estimates (β), standard errors (SE), and 95% confidence intervals (CIs) are reported for interpretability, and values were rounded to two or three decimal places as appropriate. Post hoc comparisons were conducted with *emmeans*; simple slope for categorical and continuous interactions were estimated with *emtrends* (Lenth & Piaskowski, 2025). Given that four-way interactions are hard to interpret and are prone to Type II errors due to low power, these interactions were not reported (Murayama et al., 2014). We still included them in the model to align with the experiment design. To improve optimization stability, models were estimated using the ***bobyqa*** optimizer with an increased iteration limit when necessary, and we performed model convergence checks (see supplementary materials).

Decision RT, performance accuracy, and accurate calculation RT were also analyzed. Decision RT reflects decision difficulty. Calculation accuracy and RT reflect task difficulty. Accuracy was analyzed by using the same logistic model and regressors applied in the decision analysis. Accuracy was calculated only for trials that participants accepted and performed. Trials that were rejected were excluded from the accuracy analysis (as customary for this task). Reaction times were analyzed by using a linear mixed-effects model. To control the impact of individual calculation ability on the willingness to exert effort (despite the applied calibration procedure), we fitted an additional logistic model with a calibrated accuracy rate (in the post-calibration block) as a covariate to the acceptance rate. To examine whether baseline individual differences influenced calibration, we tested correlations between calibrated response times and trait measures (depression, anxiety, and learned helplessness scores).

Finally, we examined whether depression and anxiety scores from DASS impacted the willingness to exert effort. We ran three logistic mixed-effect models on the acceptance rate data. These models included the same regressors as the previously described model, with each subclinical score added separately as an additional fixed effect. The values of the subclinical scores were standardized to z-scores. The statistical significance criteria for the post-hoc test of the slope are based on a 95% confidence interval, as per asymptotic (large sample) theory.

All the models performed can be seen in supplementary materials (Table S1). The mixed-effects model figures display the raw behavioral data. For binary outcome variables, the y-axis represents the log-odds transformation (logit scale) of the raw data (i.e., acceptance rates and accuracy) to facilitate visualization of proportional data.

### Drift diffusion model (DDM)

The drift-diffusion model belongs to a series of models called the sequential sampling models (SSMs) family. The SSMs assume that decision-making is a noisy evidence accumulation process, and choice arises once the evidence reaches one of the criterion boundaries of an option (Ratcliff & McKoon, 2008). Among all SSMs, the DDM is the most used one. The DDM posits a one-dimensional evidence accumulation process and is therefore only applicable to binary choice tasks. One significant advantage of using the DDM is that it simultaneously models both choice and RT, allowing behavioral data to be decomposed into several cognitively meaningful parameters. These include the drift rate (*v*), which determines the speed and direction of evidence accumulation; the boundary separation (*a*), which reflects the amount of evidence required for a decision; the starting point bias (*z*), which represents initial preference towards one response before stimulus presentation; and the nondecision time (*ndt*), which accounts for processes unrelated to the decision itself, including visual encoding and motor execution time.

To apply DDM to our task, similar to the previous literature of DDM in the value-based decision-making paradigm, we mapped the two decision actions (accept and reject) to the upper and lower boundaries of DDM (Guo et al., 2025; Krajbich & Rangel, 2011). To systematically examine the effect of experimental factors on DDM parameters, we did a factorial model analysis, which allows different parameters of DDM to be influenced by the experimental conditions. Specifically, we examined whether the impact of controllability on decisions is a speeding effect (i.e., an increased drift rate), a speed-accuracy tradeoff (i.e., a decreased threshold for responding), or a more goal-directed process with enhanced cognitive allocation (i.e., an increased threshold for responding).

To examine how experimental factors influenced the decision process, we implemented regression-based DDMs, in which experimental predictors were allowed to modulate specific model parameters. All predictors were effect-coded (−0.5, +0.5). Based on theoretical accounts of motivated decision-making (Berwian et al., 2020; Westbrook et al., 2020; Grahek et al., 2023). We examined how trial-level variables (reward and effort) and block-level contextual variables (controllability, reward context) influence value integration, as captured by the drift rate parameter, and decision caution, as captured by boundary separation, leading to differences in willingness to exert effort.

We compared a set of competing DDMs in which predictors were assigned to different parameters (Table 1). These included a baseline model (M0) with intercept-only parameters, (i) M1, only trial-level variables modulated drift rate, (ii) M2, only contextual variables modulated boundary separation, (iii) M3, trial-level variables modulated drift rate and contextual variables modulated boundary separation. (iv) M4, trial-level variables and contextual variables both modulated drift rate. In addition, given the interactions observed in the mixed-effect model behavioral analyses (i.e., effort × controllability and mreward × context), which showed that the contextual variables modulate the value of momentary reward or effort demand. We tested extended models, M5-7, in which contextual variables interacted with reward or effort in the drift-rate equation. Finally, we tested a model, M8 in which contextual variables also influenced the starting-point parameter, suggesting a potential pre-decisional bias.

**Table 1:** DDMs specifications: parameter mapping in competing DDMs

Model	Drift rate (*v*)	Boundary (*a*)	Starting point (*z*)
M0	Intercept	Intercept	Intercept
M1	mreward+effort+mreward:effort	Intercept	Intercept
M2	Intercept	ctrl+context	Intercept
M3	mreward+effort+mreward:effort	ctrl+context	Intercept
M4	mreward+effort+mreward:effort+ctrl+context	Intercept	Intercept
M5	mreward+effort+mreward:effort+ctrl+effort:ctrl	ctrl+context	Intercept
M6	mreward+effort+mreward:effort+context+mreward:context	ctrl+context	Intercept
M7	mreward+effort+mreward:effort+ctrl+effort:ctrl+context+mreward:context	ctrl+context	Intercept
M8	mreward+effort+mreward:effort+ctrl+effort:ctrl+context+mreward:context	ctrl+context	ctrl+context

Momentary reward (mreward), controllability (ctrl), and reward context (context)

Each DDM parameter incorporates the experiment conditions through a GLM function:

$$\theta =f({\theta}_{0}+w\times DM)$$

where

θ: Represents a DDM parameter, which can be the drift rate, starting point bias, or decision boundary.
${\theta_{0}}$: is the intercept, DM is the design matrix of the experimental conditions, and
w: is the regression coefficient capturing the effect of these conditions on the DDM parameters.

Note that since each DDM parameter operates on a different scale, we applied a suitable link function f(x) to ensure appropriate transformations. Specifically, we used the identity function for the drift rate, the exponential function for the decision boundary, and the sigmoid function for the starting point bias. For skewed RT data, we excluded the slowest and fastest 2.5% of the trials for analysis to remove outliers and to avoid over-adjustment to single fast outliers during model fitting (Peters & D’Esposito, 2020). Despite excluding extreme trials, two participants still exhibited minimum RTs below 50 ms, which constrained the *ndt* parameter to unrealistically low values for visual processing and motor execution. To address this, we applied a logit transformation to the *ndt* parameter during model fitting (Pedersen et al., 2017). It was modeled as an intercept-only parameter and constrained to remain above 50 ms via a bounded transformation to avoid implausibly short values. As *ndt* was not a parameter of primary theoretical interest, it was included to ensure stable model estimation but was not interpreted further. All effects were allowed to vary across participants, and we ran 400 iterations to ensure model convergence.

We fitted the parameters in each model for each participant using maximum likelihood estimation (MLE). We used the log-likelihood function (LLF) of the DDM, which was implemented in the R package rtdists (Singmann et al., 2026), and parameters were optimized using differential evolution in the R package DEoptim (Mullen et al., 2011). Model comparison was performed to quantitatively select the best predictive mechanistic account. We used the corrected Akaike Information Criterion (AICc) as the primary criterion, which corrects for finite-sample bias, to select the best-fitting model. In cognitive modeling studies with a relatively limited number of trials per participant and multiple free parameters, AICc provides a more reliable estimate of out-of-sample predictive performance than AIC by applying an additional penalty for model complexity (Guo et al., 2024; Li & Ma, 2021). Akaike Information Criterion was computed from the maximum log-likelihood and the number of estimated parameters, with a lower value indicating a better model fit.

The AICc and AIC equations as:

$$\begin{array}{lc}\mathrm{A}\mathrm{I}\mathrm{C}=-2 \times \mathrm{log}\mathrm{L} \left(\uptheta \right|\mathrm{y})+2 \times \mathrm{K} \\ {\mathrm{A}\mathrm{I}\mathrm{C}}_{\mathrm{c}}=-2 \times \mathrm{log}\mathrm{L} \left(\uptheta \right|\mathrm{y})+2 \times \mathrm{K} \times \left(\frac{\mathrm{n}}{\mathrm{n}-\mathrm{K}-1}\right)\\\ \ \ \ \ \ \ =\mathrm{A}\mathrm{I}\mathrm{C}+\frac{2\times \mathrm{K}\times \left(\mathrm{K}+1\right) }{\mathrm{n}-\mathrm{K}-1 }\end{array}$$

where

k: is the number of estimated parameters,
n: is the number of observations, and
L: is the maximum likelihood of the model.

AICc and BIC were reported as the sum of differences across all participants in the supplementary materials (Table S2). Parameter recovery and simulation-based model adequacy checks were conducted for the best-fitting model identified by model comparison.

To evaluate parameter identifiability, we conducted a parameter recovery analysis. Behavioral datasets were simulated using known parameter values drawn from the range of the fitted parameters. The DDM was then refitted to the simulated data using the same estimation procedure as applied to the empirical data. Recovery performance was assessed by computing the correlation between the true generating parameters and the recovered parameter estimates across simulations.

To further evaluate model adequacy, we performed simulation-based checks by generating data from the fitted parameters and comparing predicted and observed choice proportions and reaction-time distributions. Specifically, we compared predicted and observed choice proportions and reaction-time (RT) quantiles (10th, 30th, 50th, 70th, and 90th percentiles) for each response type. These comparisons allowed us to evaluate whether the model adequately captured both the distribution of RTs and the choice patterns observed in the task.

## Results

To investigate how environmental controllability and reward context influence an individual’s motivation to exert effort for rewards, we developed a novel paradigm in which controllability varied independently after each block, regardless of the total reward amount (i.e., reward context). We measured subjects’ proportion of accepting effortful trials (i.e., acceptance rate) as a primary index of their willingness to exert effort for a reward, and the response time (RT) of the choice, indexing the decision difficulty. We took the subjects’ percentage of accurate calculation in the acceptance trials (i.e., performance accuracy) as another index of effort allocation. Furthermore, we tested participants’ subjective feelings of perceived effort, perceived motivation, and awareness of changes in probability.

### Willingness to exert effort increases for high momentary rewards and low effort

**Fig. 2:**
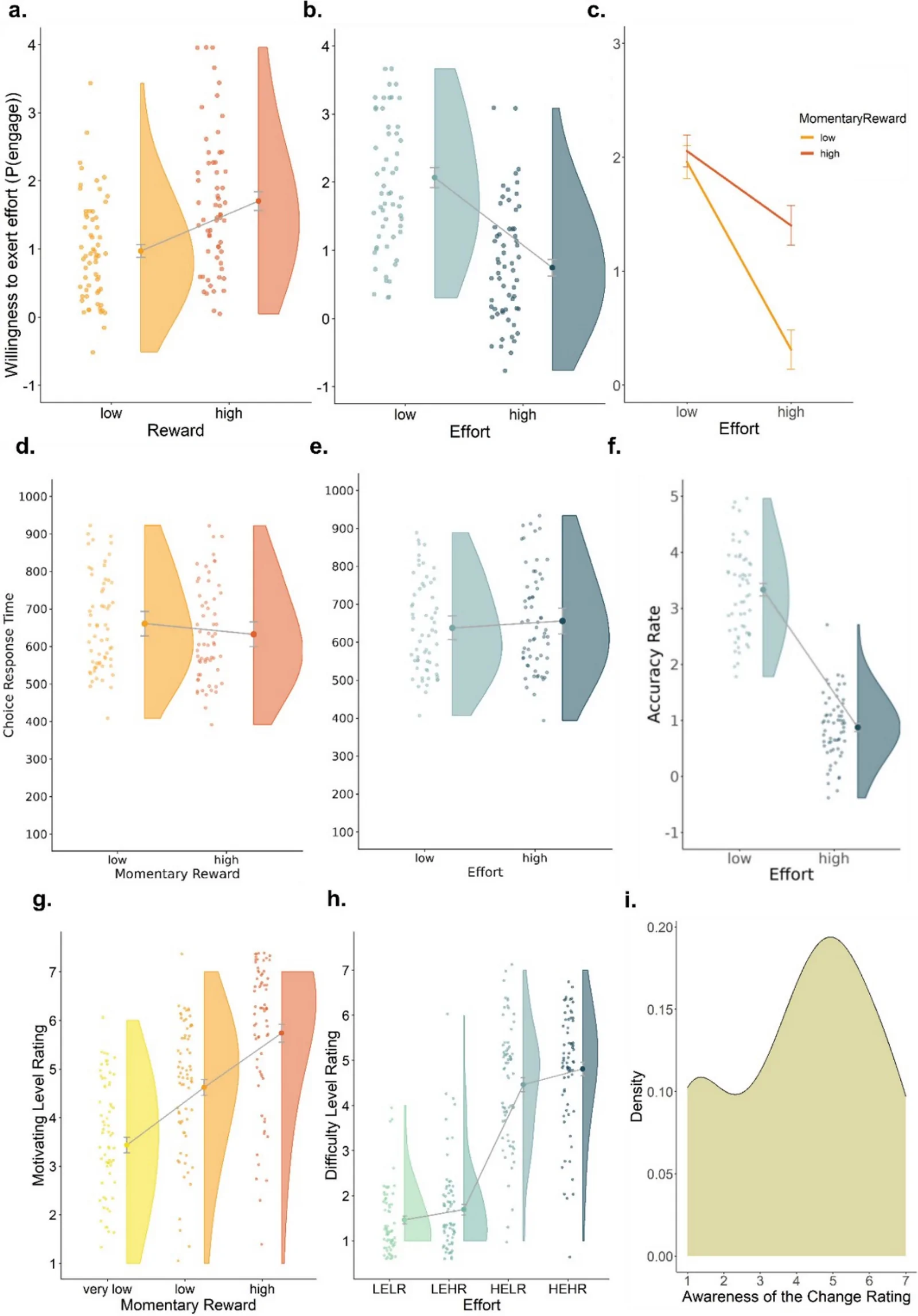
Willingness to engage in effort exertion across conditions. *Note.* **a** Effect of momentary reward on acceptance rate. **b** Effect of effort level on acceptance rate. **c** Interaction between reward and effort on acceptance rate. **d** Effect of momentary reward on choice RT. **e** Effect of effort level on choice RT. **f** Effect of effort level on accuracy. **g** Subjective rating of the extent to which participants felt motivated by momentary rewards across reward levels (very low: 30 points, low: 40 points, and high: 50 points). **h** Subjective rating of perceived effort for each cue (LELR = low effort low reward; LEHR = low effort high reward; HELR = high effort high reward; HEHR = high effort high reward). **i** Subjective rating of the extent to which participants were aware of the change in the probability of attaining an expected outcome by successful effort exertion. The error bars display ±1 within-subject standard error of the condition means. All figures display raw behavioral data unless otherwise noted. For binary outcome variables, the y-axis displays the log-odds (logit-transformed) values of the raw acceptance rate and accuracy rate

As expected, there was a significant effect of momentary reward and effort level on acceptance rate (i.e., the likelihood of accepting to engage in the effortful task, *β*_*momentary reward*_ = 0.73, SE = 0.17, χ^2^(1) = 19.90, *p* < .001;* β*_*effort*_ = −1.50, SE = 0.23, χ^2^(1) = 45.02, *p* < .001; 95% confidence intervals [CIs] [0.41, 1.05] and [−1.94, −1.06]; Figs. 2a, b). Participants engaged in effortful trials more often when a higher momentary reward was at stake and when the task at hand was easier, in line previous findings (Vassena et al., 2019b). Additionally, there was a significant reward by effort interaction, as often observed in this task (Vassena et al., 2019a), showing that the impact of reward was larger in hard trials (*β* = 0.96, SE = 0.20, χ^2^(1) = 27.48, *p* < .001, low vs. high reward | Hard : OR = 0.30, *z* = −5.03,* p* < .001, 95% CI [0.16, 0.56]; low vs. high reward | Easy: OR = 0.78, *z* = −2.23, *p* = 0.144, 95% CI [0.58, 1.05], follow-up analyses for contrasts among categorical variables are reported as odds ratios (ORs); Fig. 2c.

Decision RT analysis showed a significant main effect of effort (*β* = 0.020, SE = 0.007, χ^2^(1) = 9.36, *p* = .002) and momentary reward (*β* = −0.038, SE = 0.008, χ^2^(1) = 23.05, *p* < .001, 95% CIs [0.007, 0.034] and [−0.055, −0.023], respectively; Figs. 2d and e) on RT to decide whether to engage in effort. Participants decided faster when high momentary rewards were at stake, and slower when anticipating higher effort. Concerning calculation accuracy analysis, there was a significant main effect of effort level on performance as expected (*β* = −2.51, *SE* = 0.11, χ^2^(1) = 572.61, *p* < .001; Fig. 2f) but no significant effect of momentary reward (*β* = .01, *SE* = 0.07, χ^2^(1) = 0.02, *p* > .50, 95% CIs [−2.72, −2.31] and [−0.13, 0.15], respectively). Participants made fewer errors on easy trials than hard trials, and there was no impact of reward on accuracy.

Overall, these results confirm that the basic task manipulation worked as expected. Additionally, we asked participants to reflect on their subjective experience at the end of the experiment. Participants perceived larger reward outcomes to be more motivating*(F(2)*_motivation_ = 46.22, *p* < .001; Fig. 2d), and high effort trials as more difficult irrespective of momentary reward (*F(3)*_effort perception_ =153.27, *p* < .001; Fig. 2e). These subjective rating results confirm that our experiment successfully manipulated subjects’ perceptions of different levels of effort demand and momentary reward outcomes. Finally, participants reported that the probability that successful effort exertion led to the desired outcome varied across blocks (Fig. 2f). Participants reported at least moderate awareness of the contextual change across the task (mean awareness rating (*M* = 4.10, *t*(62) = 16.23, *p* < .001)). This also confirms that the controllability manipulation was successful.

To account for potential effects of fatigue over time, block number was standardized and added as a covariate to both the acceptance rate and accuracy models. For acceptance rate, it showed a negative effect on willingness to exert effort (*β* = −0.08, *SE* = 0.02, χ^2^(1) = 28.65, *p* < .001, 95% CI [−0.11, −0.05]), while for accuracy, it showed a positive effect on performance accuracy (*β* = 0.03, *SE* = 0.01, χ^2^ (1) = 6.95, *p* = .008, 95% CI [0.01, 0.05]). Participants were less willing to exert effort but became more accurate over time. Importantly, the key experimental effects remained significant when controlling for block number in both models predicting acceptance rate or accuracy. These suggest that the observed effects reflect task-related adaptation in willingness to exert effort and in effort allocation, rather than progressive fatigue throughout the experimental task.

Additionally, we tested correlations between calibrated response times and trait measures (depression and anxiety scores) to examine baseline individual differences that may have influenced calibration. These analyses revealed no significant associations between calibrated response speed and depression or anxiety scores (depression: *r* = 0.08, *t*(59) = 0.64, *p* = 0.5; anxiety: *r* = 0.07, *t*(59) = −0.60, *p* = 0.58). These suggested that there were no baseline individual differences in how people were calibrated on our task.

### Willingness to exert effort dynamically adjusts to environmental controllability

**Fig. 3:**
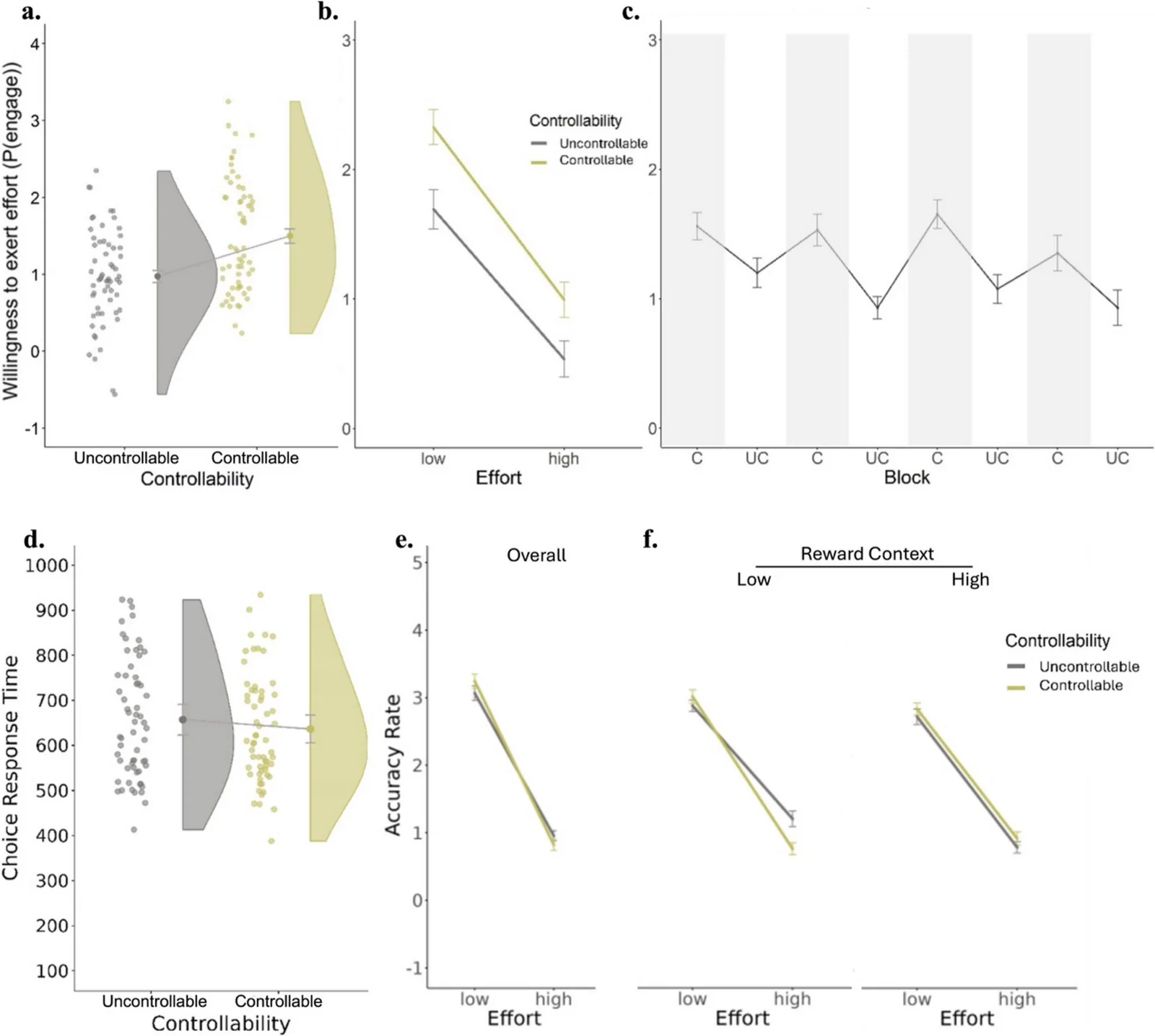
The impact of controllability on willingness to exert effort. *Note.* **a** Participants accepted to engage in effortful trials more often in controllable conditions (compared with the uncontrollable). **b** The increase in willingness to exert effort in controllable blocks is more pronounced for easy trials (as compared to hard trials). **c** The willingness to exert effort dynamically adjusted to the varying environmental controllability across blocks. The error bars display ±1 within-subject standard error of the condition means

We examined how individuals’ motivation adjusts to environmental controllability. As hypothesized, subjects showed a higher willingness to exert effort for reward in controllable blocks than in uncontrollable blocks (*β* = 0.80, *SE* = 0.09, χ^2^(1) = 80.54, *p <* .001, 95% CI [0.63, 0.98], Figs. 3a, c). This indicated that subjects adjusted their willingness to exert effort according to the variation in environmental controllability across blocks. Moreover, we found a significant interaction between effort and controllability (*β* = −0.62, *SE* = 0.14, χ^2^(1) = 28.47, *p* < .001, 95% CI [−0.85, −0.39]; Fig. 3b). Participants were more sensitive to effort level in controllable blocks than in uncontrollable blocks (easy vs. hard | Controllable: OR = 6.14, *z* = 7.21, *p* < .001, 95% CI [3.17, 11.90]; easy vs. hard | Uncontrollable: OR = 3.29, *z* = 5.69, *p* < .001, 95% CI [1.90, 5.72]). Thus, controllability amplified sensitivity to effort demands participants were more willing to exert effort for easy trials (relative to hard trials) in controllable blocks.

The decision RT analysis revealed a significant main effect of controllability on RT to decide whether to exert effort (*β* = −0.026, *SE* = 0.007, χ^2^(1) = 14.24, *p* < .001, 95% CI = [−0.040, −0.012]; Fig. 3d). Participants were faster in making decisions in the controllable blocks. In the accuracy rate analysis, we found a significant interaction effect between effort and controllability (*β* = −0.36, *SE* = 0.14, χ^2^(1) = 6.47, *p* = .011, 95% CI [−0.64, −0.08]) and a significant three-way interaction among effort, controllability, and reward context (*β* = 1.08, *SE* = 0.28, χ^2^(1) = 14.56, *p* < .001, 95% CI [0.52, 1.63]; Figs. 3e, f). Across reward contexts, the impact of difficulty (effort level) on accuracy is greater in controllable than in uncontrollable blocks. Participants made fewer errors on easy trials than on hard trials in both conditions, but the difference was larger in the controllable blocks (easy vs. hard | Controllable: OR = 14.8, *z* = 21.37, *p* < .001, 95% CI [11.56, 19.00]; easy vs. hard | Uncontrollable: OR = 10.3, *z* = 18.36, *p* < .001, 95% CI [8.05, 13.30]). Importantly, this effort x controllability interaction was modulated by reward context. This interaction was present in the low-reward contexts (*β* = 0.88, *SE* = 0.08, *p* < .001, 95% CI [0.17, 0.63]), but not in high-reward contexts (*β* = −0.12, *SE* = 0.09, *p* > .05, 95% CI [−0.35, 0.10]). To clarify, in low reward context, on hard trials participants performed significantly better in uncontrollable compared to the controllable blocks (Hard trials: Controllable vs. Uncontrollable | Low Reward Context: OR = 1.50, *z* = 4.44, *p* < .001, 95% CI [1.18, 1.89]), whereas on easy trials they performed significantly better in controllable than uncontrollable blocks (Easy trials: Controllable vs. Uncontrollable | Low Reward Context: OR = 0.61, *z* = −2.64, *p* = .04, 95% CI [0.38, 0.99]).

### Willingness to exert effort depends on both momentary reward and reward context

**Fig. 4:**
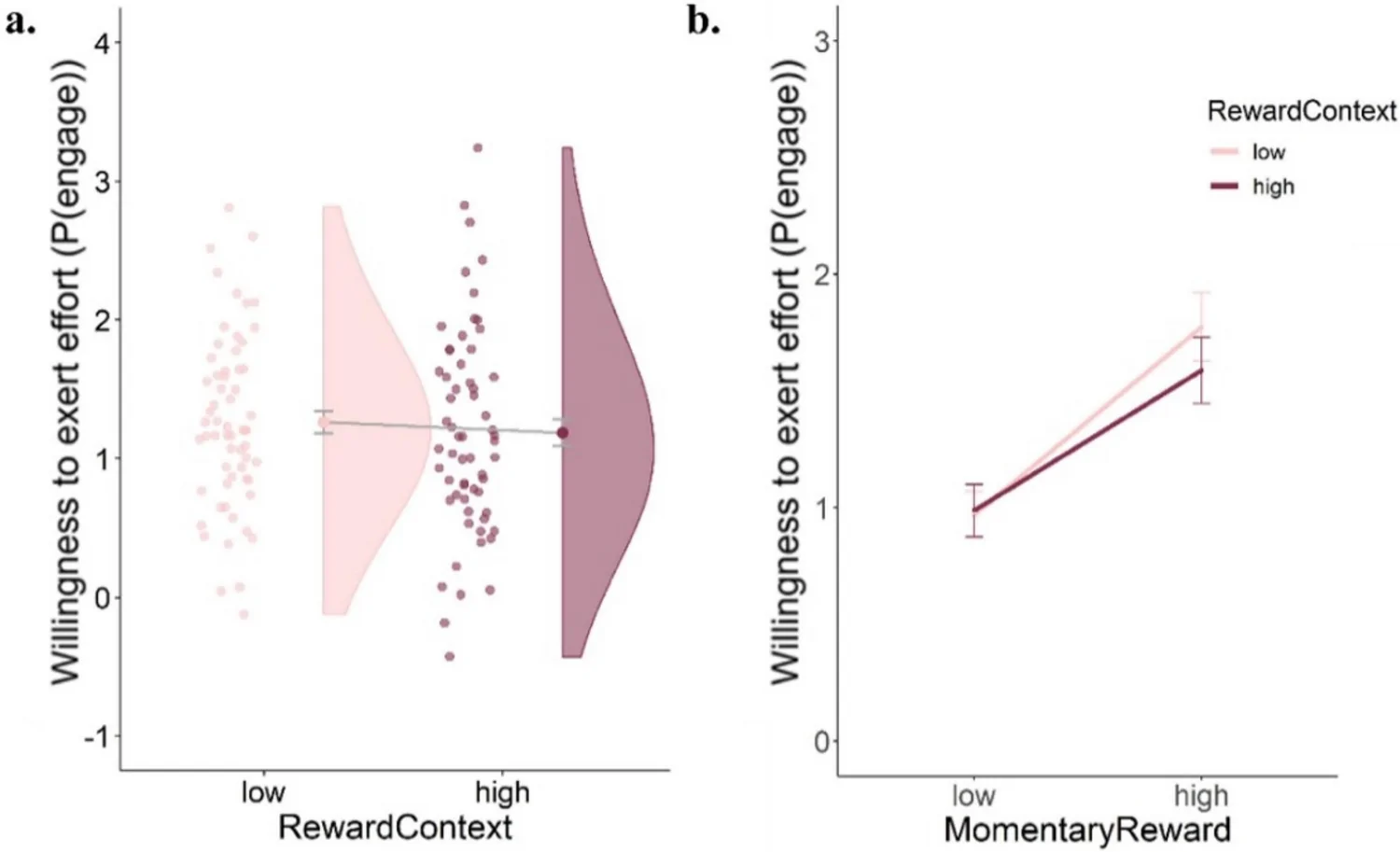
The impact of reward context on willingness to engage in effort. *Note.* **a** No main effect of reward context on the willingness to exert effort. **b** Participants accepted exerting more effort for high momentary rewards in low-reward contexts, showing that the value of the reward on offer is scaled to the context. The error bars display ±1 within-subject standard error of the condition means

Individuals’ willingness to exert effort to gain momentary rewards varied depending on the reward context, as indicated by a significant interaction between momentary reward and reward context (*β* = −0.36, SE = 0.16, χ^2^(1) = 6.85, *p* = .009, 95% CI [−0.63, −0.09]; Fig. 4b). In the low-reward context, momentary reward had a larger impact on willingness to exert effort (low vs. high | low RC: OR = 0.40, *z* = −4.73, *p* <.001, 95% CI [0.28, 0.59]). Conversely, in high-reward contexts, the impact of momentary reward on willingness to exert effort was smaller (low vs. high | high RC: OR = 0.58, *z* = −3.42, *p* =.001, 95% CI [0.42, 0.79]). Thus, subjects exerted more effort for high momentary rewards in low reward context, indicating that they tracked the average reward rate in the environment and integrated it into the valuation of momentary rewards. There was no significant main effect of reward context (*β* = −0.09, *SE* = 0.11, χ^2^(1) = 0.67, *p* = .414, 95% CI [−0.31, 0.13]; Fig. 4a). The interaction effect remained significant when the individual calibrated calculation accuracy rate was added as a covariate in the model. This indicates that subjects’ valuation of momentary rewards depends on the reward context and is reliable, irrespective of individual differences in calculation performance.

In the RT analysis, we found no significant effect of reward context on decision-making RT. We observed a significant interaction between effort and reward context on decision RT (*β* = .03, *SE* = 0.01, χ^2^(1) = 4.21, *p* = .040, 95% CI [0.001, 0.05]). Participants were slower to accept engaging in high-effort trials in the high-reward context (compared to the low-reward context). Regarding accuracy, participants made more errors in the high-reward context than in the low-reward context (*β* = −.14, *SE* = 0.07, χ^2^(1) = 4.68, *p* = .031, 95% CI [−0.27, −0.01]).

### People with higher depression levels are more sensitive to controllability in high reward contexts

We hypothesized that individuals with higher levels of subclinical psychopathology traits, such as depression and anxiety, may be more affected by lower levels of controllability. We predicted that higher levels of depressive traits are associated with reduced willingness to exert effort for reward in an uncontrollable environment. This is based on prior evidence showing that patients with depression tend to expend less effort to gain rewards (Treadway et al., 2012; Horne et al., 2021) and that uncontrollable environments may induce learned helplessness (Maier & Seligman, 2016). Conversely, we hypothesized that individuals with higher levels of anxiety would show increased willingness to exert effort for rewards in an uncontrollable environment, as anxiety has been linked to excessive avoidance of punishment, even in safe situations (Sharp et al., 2022).

**Fig. 5:**
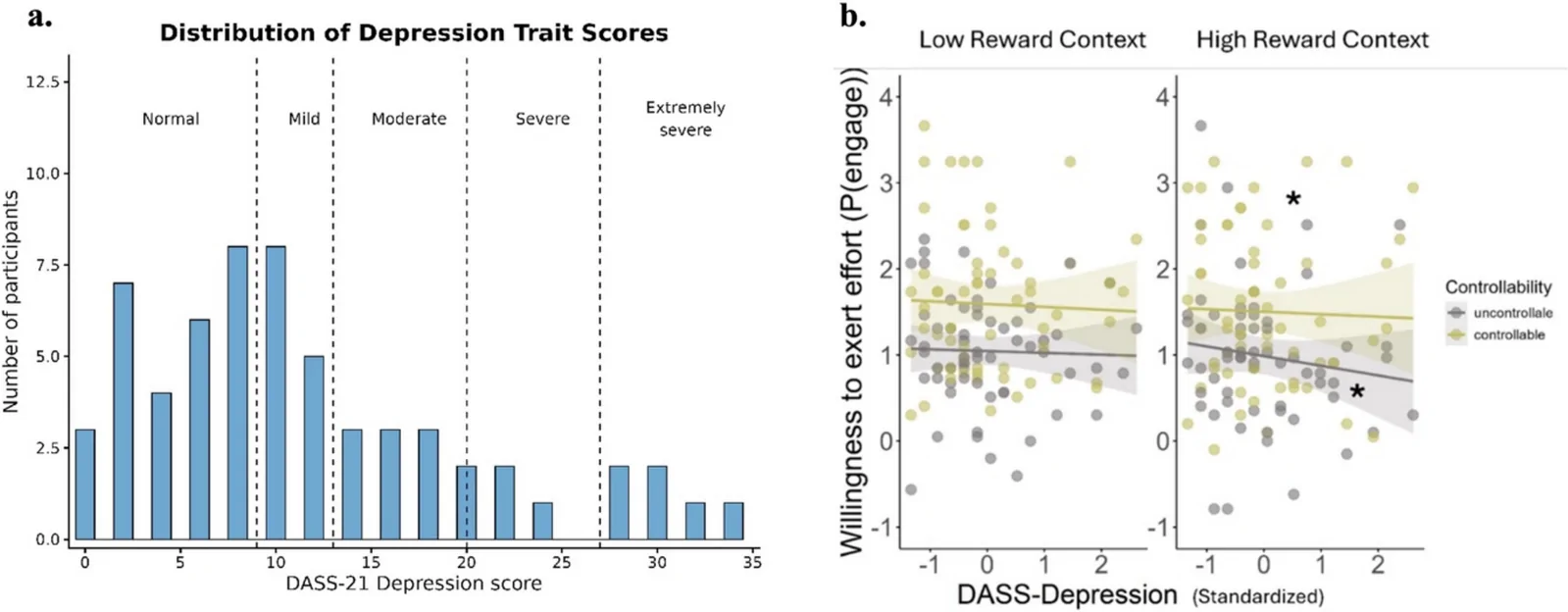
The effect of subclinical traits of depression on willingness to engage in effort exertion depends on controllability and reward context. *Note*. **a** Distribution of participants’ DASS-21 depression scores. Dashed vertical lines indicate the conventional DASS severity thresholds (normal, mild, moderate, severe, and extremely severe). **b** Interaction among DASS depression scores, controllability, and reward context on acceptance rate. *p < .05

Depression scores showed substantial variability (*M* = 11.54, *SD* = 8.60, Normal (0–9): *n* = 28, Mild (10–13): *n* = 13, Moderate (14–20): *n* = 11, Severe (21–27): *n* = 3, Extremely Severe (>27): *n* = 6; Fig. 5a), 55.1% participants’ score were above the cutoff and spanned multiple severity levels according to DASS guidelines.

We observed a significant three-way interaction between depression levels, controllability, and reward context (*β* = 0.33, *SE* = 0.14, χ^2^(1) = 5.44, *p* = 0.020, 95% CI [0.05, 0.61]; Fig. 5). In the high reward context, people with higher subclinical depression levels were more sensitive to controllability (*β*_*uncon: con | high rc*_ = −0.28, SE = 0.13, *p* = .034, 95% CI [−0.54, −0.02]) and showed decreased willingness to exert effort in uncontrollable blocks. Conversely, individuals with lower subclinical depression levels kept exerting effort to get more reward even in uncontrollable situations (*β*_uncontrol*high rc_ = −0.27, *SE* = 0.13, *p* = .041, 95% CI [−0.54, −0.01]). This pattern was not observed in low reward contexts (*β*_*uncon: con | low rc*_ = 0.05, *SE* = 0.09,* p* = .600, 95% CI [0.52, 0.60]). All remaining effects were not significant (all *p* > .1), and there were no significant effects of anxiety.

There was no significant main effect of subclinical depression levels (*β*_depression_= −.12, *SE* = 0.11, χ^2^(1) = 1.19, *p* = .276, 95% CI [−0.35, 0.07]) or anxiety levels (*β*_anxiety_ = −.02, *SE* = 0.11, χ^2^(1) = 0.89, *p* = .836, 95% CI [−0.19, 0.23]).

## Drift diffusion modeling results

### Drift rate is modulated by reward, effort, controllability, and reward context, whereas decision boundary is selectively modulated by controllability

Model comparison (AICc) indicated that Model 7, which included both trial-wise variables (momentary reward and effort) and block-wise variables (controllability and reward context) in the drift-rate parameter and block-wise variables in the boundary parameter, provided the best fit to the data, comparable with Model 4 (which included only drift rate being influenced by all factors) and Model 8 (which additionally included block-wise predictors in the starting-point parameter).

**Fig 6:**
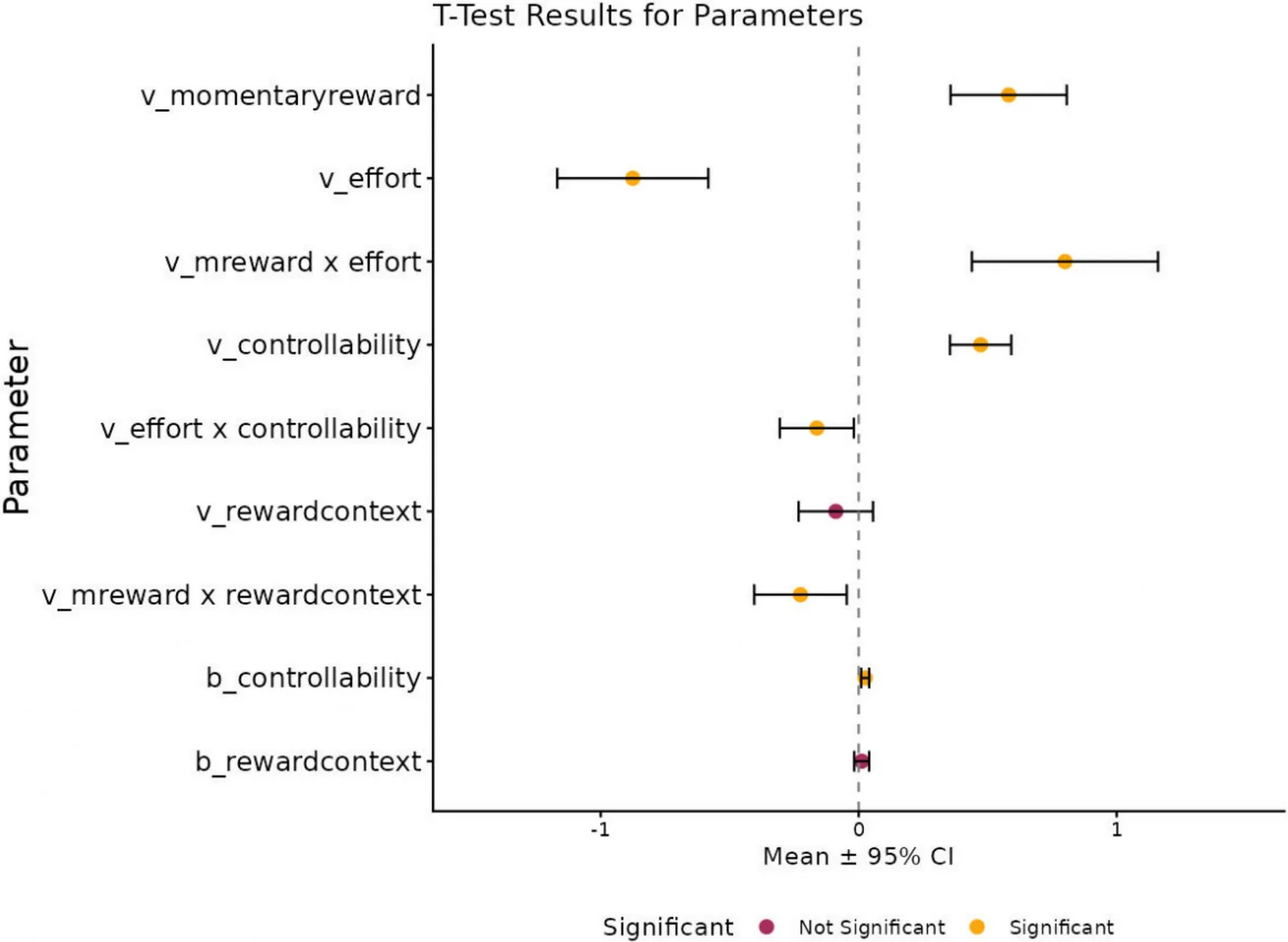
Parameter estimation from the DDM. *Note.* Latent parameter estimates from the model, with the statistical test result. CI = confidence interval. Simulation-based predictive checks on the observed and simulated data on choice proportions, and choice RT quantiles (see supplementary materials Figs. S1-S2). Parameter recovery validates model estimation of the parameters (see supplementary materials Fig. S3). The identity function was used to fit the drift rate, the exponential link function to fit the decision boundary, and the results were plotted on the original scale

Aligned with the (G)LMM results, momentary reward, and controllability had a positive effects on drift rate (*t*(60) _momentary reward_ = 5.16, *p* < .001, *M* = 0.58, d = 0.66; *t*(60) _controllability_ = 7.96, *p* < .001, *M* = 0.47, d =1.02), as well as the momentary reward and effort level interaction (*t*(60)_momentary reward: effort_ = 4.43, *p* < .001, *M* = 0.80, d = 0.57), whereas effort level had a negative effect on drift rate (*t*(60) effort = −5.98, *p* < .001, *M* = −0.88, d = −0.77), as well as the effort and controllability interaction (*t*(60)_effort: controllability_ = −2.26, *p* = .03, *M* = −0.16, d = −0.29) and momentary reward and reward context interaction (*t*(60)_momentary reward: reward context_ = −2.52, *p* = .01, *M* = −0.023, d = −0.32) 95% CIs [0.36, 0.81], [0.35, 0.59], [0.44, 1.16], [−1.17, −0.58], [−0.31, −0.02], and [−0.41, −0.05], respectively; Fig. 6). A positive reward × effort interaction indicated that the positive impact of reward on value integration was larger at higher effort levels, consistent with integrated cost–benefit valuation. A negative effort × controllability interaction indicated that the impact of effort on drift rate was reduced in controllable conditions, suggesting that effort demand is weighted less strongly when outcomes are under participants’ control. The negative momentary reward × reward context indicated that the impact of momentary reward on drift rate was reduced in a high reward context.

We also observed a larger boundary (decision threshold) in controllable conditions than in uncontrollable conditions (*t*(60) _controllability_ = 3.21, *p* = .002, *M* = 0.02, d = 0.41), but not in the different reward contexts (*t*(60) _rewardcontext_ = 0.80, *p* = .425, *M* = 0.01, d = 0.10, 95% CIs [0.01, 0.04] and [−0.02, 0.04]). There were no significant effects on the starting point (*p* > .1). In summary, participants exhibited a faster drift rate, accompanied by higher decision threshold in controllable blocks, which was inconsistent with explanations involving a speeding effect (i.e., increased drift rate) or a speed-accuracy tradeoff (i.e., decreased threshold for acceptance).

The model successfully reproduced both acceptance rates and RT distributions, indicating a good fit to the behavioral data. Recovery correlations were high for the primary drift-rate and boundary parameters (*r* ≈ 0.75–0.9), indicating reliable estimation of the main model parameters.

## Discussion

The controllability and richness of the environment are two key factors that drive human motivation to allocate effort to obtain a desired outcome. We investigated the effect of varying controllability and reward context on motivation to exert mental effort. Three major findings stem from this study: First, participants adjusted their motivation in response to changes in controllability by reducing their willingness to engage in effort during uncontrollable blocks and increasing it again in controllable blocks. Drift Diffusion Modeling revealed that motivation adaptation was driven by changes in value difference integration and decision caution rather than a simple speed-accuracy tradeoff. Second, participants tracked the reward context and integrated it with momentary rewards, as evidenced by a higher willingness to exert effort for high momentary rewards, particularly in the low-reward context. Third, importantly, the effects of controllability varied with subclinical depression as a function of reward context. When the environment was rich, individuals with lower depression levels kept on exerting effort to get more reward, even when it became uncontrollable. Conversely, in the same conditions, individuals with higher subclinical depression showed diminished willingness to exert effort. These findings bring a novel understanding of how people adapt effort exertion to varying controllability and reward contexts and indicate a candidate cognitive mechanism for increased susceptibility in subclinical depression.

The main goal of this study was to investigate the impact of controllability, reward context, and their interplay on the willingness to exert effort to obtain rewards. Previous studies have generally not accounted for mental effort nor systematically varied the average reward rate and controllability within the same design. Our CoMET paradigm allowed us to examine the impact of controllability and reward context on motivation adjustment separately and systematically by not only dissociating but also manipulating the average reward rate and controllability while maintaining constant predictability. Importantly, and as hypothesized, participants calibrated their effort allocation to the controllability of the environment by accepting engaging and exerting effort more often in controllable blocks than in uncontrollable blocks. This is conceptually in line with the *efficacy perspective* (Grahek et al., 2023), where higher expected efficacy leads to increased control allocation (Frömer et al., 2021; Yang & Delgado, 2025). Our results extend this principle to effort-based decision-making, showing that people dynamically adapt their decisions to exert effort in response to changes in controllability. Participants also responded more quickly during decision-making in controllable blocks.

Our findings are in line with the view that estimated controllability determines the balance between proactive and reactive responses (Moscarello & Hartley, 2017). Our participants learned that controllability varied across blocks and calibrated their willingness to engage in the task, accordingly, displaying more proactive responses (i.e., engaging in effort and exerting instrumental control) in the controllable blocks and more reactive responses (i.e., refusing to engage in effort) in the uncontrollable blocks. Thus, our study is consistent with previous research on proactive and reactive control modes and further extends this framework to effort-based decision-making and effort allocation.

It is important to highlight some conceptual differences from prior work. We focused on objective rather than subjective controllability by examining decisions to invest effort in comparison to ratings of perceived control (Grahek et al., 2023; Teodorescu & Erev, 2014). Additionally, we focus on controllability, intended as the likelihood of obtaining the desired outcome upon successful effort exertion. This implementation aligns with the concept of performance efficacy but does not consider the concept of control efficacy, specifically whether the deployed control successfully leads to accurate performance (Grahek et al., 2023; Shenhav et al., 2021). Yet, our task is preceded by a calibration procedure to make difficulty levels comparable across participants, thus leading to similar levels of control efficacy. Although average accuracy is kept constant across participants, it is possible that a higher difficulty level, naturally leading to lower accuracy, may elicit a perception of relatively lower control. This hypothesis could be tested in a future study that includes both decision-making and subjective ratings.

The current implementation of controllability diverges from the *Information-theoretic approach* (Ligneul et al., 2022) where controllability is estimated based on whether actions influence state transitions (in the absence of reward). This approach focuses on isolating the causal relationship between actions and states without considering anticipated effort exertion or reward expectations. This approach models beliefs about the controllability of an environment based on the extent to which actions influence state transitions, independently of reward. Thus, although action–state contingencies provide the information used to infer controllability, the resulting estimate concerns the broader controllability of the environment rather than the controllability of a particular task-relevant action. We diverge from this view, aligning with previous literature on perceived control that suggests that multiple variables can influence the perception and inference of controllability, including instrumental contingency, average reward rate (Ly et al., 2019), reward frequency (Teodorescu & Erev, 2014), action and outcome associations (Khalighinejad & Haggard, 2016), and prior control beliefs (Huys & Dayan, 2009). In line with a definition by Huys and Dayan (2009), our work operationalizes controllability as the probability of achieving the desired outcome upon successful effort exertion. In our study, the impact of controllability on motivated behaviour is approached from a constructivist *reinforcement meta-learning* perspective (Vassena & Gottlieb, 2025), where individuals learn and integrate decision-relevant variables, such as required effort, probability of success, reward context, and environmental volatility to optimize behavioural selection (Silvestrini et al., 2023; Silvetti et al., 2018). Hereby, quantifying variations in all relevant motivational variables is essential to understanding motivation to exert effort.

As hypothesized, decisions to invest effort were also influenced by reward context, as observed in the momentary reward by reward context interaction. Participants were more willing to engage in effortful trials for high momentary rewards in low-reward contexts, indicating that outcome value was evaluated relative to the reward environment. This pattern is consistent with the value normalization account (Palminteri & Lebreton, 2021), whereby the same rewards are perceived as more valuable when encountered in poorer contexts. This aligns with effects of reward context previously observed in trial-by-trial performance adjustments across very different tasks and metrics (such as RT in task-switching paradigms (Otto & Vassena, 2021)). Importantly, our findings extend previous work by showing the impact of this contextual scaling on decisions to engage in effort, rather than only on effortful performance.

In contrast, our results are less consistent with an pure opportunity cost of time explanation, where a high reward environment would invigorate faster responses at the cost of accuracy (Boureau & Dayan, 2011; Niv et al., 2007). Recent studies have shown that the average reward rate modulates effortful cognitive control across different tasks, including the Simon task (Lin et al., 2022) and the Flanker task (Devine et al., 2021). Hereby, higher reward rates were associated with more errors, especially in more difficult conditions, albeit without faster responding. This can be interpreted as a withdrawal of costly cognitive processing when the average reward rate is high (Lin et al., 2022), rather than speeded motor responses due to invigoration or impulsivity. Translated to our task manipulation, an opportunity cost account would predict higher willingness to engage in more effortful trials (in decisions), faster responses, and worse performance accuracy in richer environments. However, we found no reward context effect on decisions to exert effort, nor an overall speeding effect on choice RT. Although participants were sensitive to the momentary reward manipulation, as reflected in significant main effects of reward on both choice behaviour and choice response time, the reward × reward context interaction was observed for choices but not for choice response time. This suggests that response-time measures may have been less sensitive to context-dependent reward modulation in the present task. Moreover, participants were slower, rather than faster, to accept high-effort trials in a high-reward context. Broadly consistent with prior effortful cognitive control work, our findings show reduced performance accuracy in the high-reward context, without faster responses. Taken together, these results are better explained by contextual modulation of subjective value rather than by an increase in response speed driven by the opportunity cost of time.

Sensitivity to reward context may also largely vary across individuals, as we observed substantial variability in decision-making regarding the context. Another potential account for the variability regarding the context effect is that our task involved combining decision-making and performance: high reward availability might have boosted people’s positive affect, encouraging them to exert effort (van Steenbergen et al., 2021), perhaps optimistically above their ability, leading to disengagement during performance. Some individuals may exert more effort for high rewards in poor environments (i.e., value-normalization), while others may experience a more general boost (van Steenbergen et al., 2021), showing increased willingness in rich environments (i.e., driven by higher opportunity costs or increased positive affect) followed by poorer performance not keeping up with decisions. These possibilities remain to be investigated. Overall, our findings favored the value normalization account rather than an opportunity cost explanation.

Concerning the underlying cognitive mechanism, the DDM analysis of choice and RT data revealed that willingness to exert effort adapted to high momentary reward, low effort demand, and controllability via more efficient value integration and a higher decision threshold in controllable blocks. This is conceptually consistent with previous work linking higher drift rates with larger rewards, higher performance efficacy during effortful performance (Grahek et al., 2023; Leng et al., 2021; Yee et al., 2024) and lower drift rate associated with high effort choice (Berwian et al., 2020; Westbrook et al., 2020). The DDM results further revealed a negative reward × reward context interaction in drift rate, indicating that the impact of momentary reward (i.e., faster drift rate) was attenuated in richer reward environments. It is, however, inconsistent with a lower information accumulation rate and decision threshold in high average reward rate environments, although this effect was observed on performance rather than decisions (Otto & Daw, 2019).

Importantly, we also observed a higher decision threshold in controllable blocks. While boundary separation is often interpreted as a response caution, in the context of value-based decisions, it more specifically reflects an evidence requirement of relative value before committing to a choice (Tajima et al., 2016). The higher threshold in controllable blocks, therefore, suggests that participants required a larger value difference between the two options before committing to effort allocation and engaged in a more deliberative evaluation process when their performance affected outcomes.

This interpretation aligns with frameworks proposing that cognitive control allocation reflects a cost–benefit computation, in which individuals allocate effort when exerting control is expected to be worthwhile or efficacious (Kool et al., 2010, 2017; Shenhav et al., 2013; Vriens et al., 2025). Under these circumstances, participants may allocate more cognitive resources to evaluating the costs and benefits of exerting effort before committing to a decision. It is also consistent with the view that controllability promotes instrumental over Pavlovian control systems—controllability increases the recruitment of goal-directed control processes and reduces reliance on more automatic or Pavlovian response tendencies (Dorfman & Gershman, 2019; Grahek et al., 2023; Ligneul et al., 2022). While previous work has focused on performance or predictions of state transitions, our study demonstrates that these motivational influences apply to information integration during effort-based decision-making, preceding effort allocation. In the present task, controllability may have encouraged participants to allocate more cognitive resources to evaluating the costs and benefits of exerting effort before committing to a decision. In this sense, the threshold effect likely reflected a more goal-directed process with enhanced cognitive control during the integration process than a speed-accuracy tradeoff or simply reduced impulsivity. Hence, people applied strategic resource optimization by allocating more control in both integration and decision-making processes in the controllable blocks.

While previous work has focused on performance, our study demonstrates that these motivational influences also apply to information integration during decision-making. The higher threshold in controllable blocks indicated more caution and less impulsivity when deciding to exert effort. This likely reflected a more goal-directed process with enhanced cognitive control during the integration process than a speed-accuracy tradeoff or a merely speeding effect. Hence, people applied strategic resource optimization by allocating more control in both integration and decision-making processes in the controllable blocks. This is consistent with the view that controllability promotes instrumental over Pavlovian control systems, extending it to the domain of effort-based decision-making (Dorfman & Gershman, 2019; Grahek et al., 2023; Ligneul et al., 2022).

The individual differences analysis showed a negative association between subclinical depression and the willingness to exert effort in uncontrollable and high-reward contexts. This is consistent with impaired motivation observed in people with depression, who display decreased sensitivity to reward and are more susceptible to experiencing a lack of control (Dulin et al., 2013; Grahek et al., 2019; Voulgaropoulou et al., 2020). Importantly, we find that in subclinical depression, motivational impairment only becomes evident in high-reward contexts. In rich environments, when things become uncontrollable, people with higher depression levels tend to disengage more, while people with lower depression scores keep on exerting effort. This finding points towards a potential mechanism by which subclinical vulnerability to depression may be detected, particularly in effortful yet still positive environments. Speculatively, this may offer insights into possible resilience mechanisms: trying less hard in environments that are currently rich may penalize individuals later on, as they will be less readily able to track upcoming and potentially critical environmental changes. This aligns with the learned helplessness framework and suggests that subclinical depression may be associated with impaired calibration of effort allocation.

From a methodological point of view, our novel CoMET paradigm carries some advantages and limitations. This task systematically varies controllability and reward context while controlling for the average reward rate and maintaining predictability constant. Through the yoking procedure, average reward rate in uncontrollable blocks was matched to average reward rate in controllable blocks: larger reward amount from bonus reward trials compensated for the lower amount of momentary reward that could be obtained through regular trials. Thus, uncontrollable blocks systematically yield similar overall reward rates as controllable blocks. In addition, reward context was manipulated orthogonally to controllability, allowing overall reward richness to vary independently of controllability. A remaining design limitation is that the average reward rate is controlled in a block-wise manner, but not at the trial level. This choice is based on our framing of controllability as constructed by the integration of motivationally relevant variables (reward, effort, probability), and aligns with previous instantiations of controllability (Huys & Dayan, 2009). In this perspective, trial-by-trial instrumental value is a key driver of effort exertion and differs across controllability conditions manipulated in the task. This can be considered a limitation of this framing, compared, for example, with the information-theoretic approach, where controllability is estimated fully independently from incentive (Ligneul et al., 2022). Additionally, while the reward amount is controlled, rewarding outcome frequency is lower in uncontrollable blocks than in controllable ones, as the probability to attain a reward by exerting effort is lower. These limitations reflect the challenge of separating reward-related influences from the concept of control in instrumental behaviour and indicate further need for comparative tasks attempting to dissociate these aspects.

An important avenue for future work concerns the sensitivity to reward context. Although current findings support a value normalization account, it remains to be specified which normalization rule can better explain the modulation of motivated behavior (i.e., the divisive normalization rule (Louie et al., 2013) vs. the range normalization (Bavard & Palminteri, 2023; Rustichini et al., 2017)). Additionally, individuals may vary significantly in the extent to which they resort to a value normalization strategy (exerting more effort for high rewards in poor environments) or be influenced by the average reward rate more generally (simply exerting more effort in high-reward environments). Furthermore, explicitly testing decisions to exert effort when the opportunity cost of time is relevant (i.e., with a variable number of trials) may reveal further nuances in behavior. Finally, future studies with a large sample size should further explore the association between subclinical traits and these incentive factors.

## Conclusions

Our study provides a new systematic framework underscoring the pivotal roles of controllability and reward context in shaping human motivation to exert effort. This highlights the importance of understanding motivated decisions from a constructivist perspective, where multiple environmental and individual variables are dynamically integrated to adaptively guide motivated behaviour. These findings are particularly relevant for identifying environmental conditions that may exacerbate susceptibility in more vulnerable individuals, such as those with higher subclinical depression. Overall, this study offers a valuable framework for characterizing motivational impairments and may open novel pathways for mitigating motivation deficits in stress-related disorders.

## Supplementary Information

Below is the link to the electronic supplementary material.

Supplementary file1 (PDF 892 KB)

## Data Availability

Raw research data is available through the OSF link (https://osf.io/3t4kr/overview) in the file section.

## Code availability

Analysis code is available through the OSF link (https://osf.io/3t4kr/overview) in the file section.

## Declarations

### Additional information

T.V. is a PhD student enrolled in the National PhD in Artificial Intelligence, XXXVII cycle, course on Health and Life Sciences, hosted by Università Campus Bio-Medico di Roma.
