# Supplementary material for: Motivation to exert effort dynamically adapts to reward context and environmental controllability
Source: Cogn Affect Behav Neurosci. 2026 Aug 31;26(5):1983–2003. doi: 10.3758/s13415-026-01484-4 (PMC13615067; doi:10.3758/s13415-026-01484-4)
Supplement: Supplementary file 1 [file 13415_2026_1484_MOESM1_ESM.pdf]

Motivation to Exert Effort Dynamically Adapts to Reward Context and Environmental Controllability

Table S1.

(Generalized) Linear mixed-effect model specification.

Model	Fixed effect structure	Random effect structure	Dependent variable
M1	Reward*Effort*Ctrl*Context	1+Reward*Effort*Ctrl *Context | ID	Acceptance rate
M2	Reward*Effort*Ctrl*Context+calibrated calculation accuracy rate	1+Reward*Effort*Ctrl *Context | ID	Acceptance rate
M3	Reward*Effort*Ctrl*Context+block-number_z	1+Reward*Effort*Ctrl *Context | ID	Acceptance rate
M4	Reward*Effort*Ctrl*Context+ Context_order	1+Reward*Effort*Ctrl *Context | ID	Acceptance rate
M5	Reward*Effort*Ctrl*Context	1+Reward*Effort*Ctrl *Context | ID	Choice RT
M6	Reward*Effort*Ctrl*Context	1+Reward*Effort*Ctrl *Context | ID	Accuracy
M7	Reward*Effort*Ctrl*Context+block-number_z	1+Reward+Effort+Ctrl +Context | ID	Accuracy

GLMM Model Convergence Checks.

Model convergence was assessed by inspecting optimizer warnings and the gradient information returned by the model-fitting procedure (Bates et al., 2015). All models were estimated using the **bobyqa optimizer** and converged successfully with no convergence warnings. We assessed potential singular fits using the isSingular() function in the **lme4** package. Some models, including those predicting choice response time (RT) and performance accuracy, produced singular fit warnings, indicating that certain random-effect variance components were estimated close to zero and that the corresponding random slopes were not strongly supported by the data. Importantly, model convergence was assessed by inspecting optimizer convergence codes and gradient diagnostics. All models returned a convergence code of 0 and showed no warnings indicating non-convergence. Therefore, the specified random-effects structure was retained.

Residual diagnostics were evaluated using simulation-based methods implemented in the DHARMa package (Hartig, 2016). For the choice RT models, the Kolmogorov–Smirnov test indicated minor deviations from uniformity. However, visual inspection of the residual distribution for choice RT data and a binned residual plot for Accuracy data (i.e., binary data) suggested that the overall model fit was appropriate (Gelman et al., 2000). Tests for dispersion and outliers were non-significant, and residuals showed no systematic relationship with model predictions.

Potential multicollinearity among predictors was assessed using variance inflation factors (VIF) computed with the performance package. All VIF values were below 2, indicating low multicollinearity. Overall, these diagnostic checks indicate that the mixed-effects models were well specified and that the reported results are unlikely to be driven by convergence issues or violations of model assumptions.

Table S2.

AICc values for model comparison

Model	AIC_total	AICc_total	delta_AIC	delta_AICc
**M0**	5656	5664	4356	3141
**M1**	2014	2037	783	729
**M2**	5566	5583	4335	4275
**M3**	1914	1951	682	643
**M4**	1381	1419	150	111
**M5**	1523	1578	291	270
**M6**	1612	1668	381	360
**M7**	1231	1308	0	0
**M8**	1302	1403	70.3	95.4

Figure S1.

Simulation-based predictive checks on the observed and simulated data on choice proportions

a. *Reward x effort interaction*

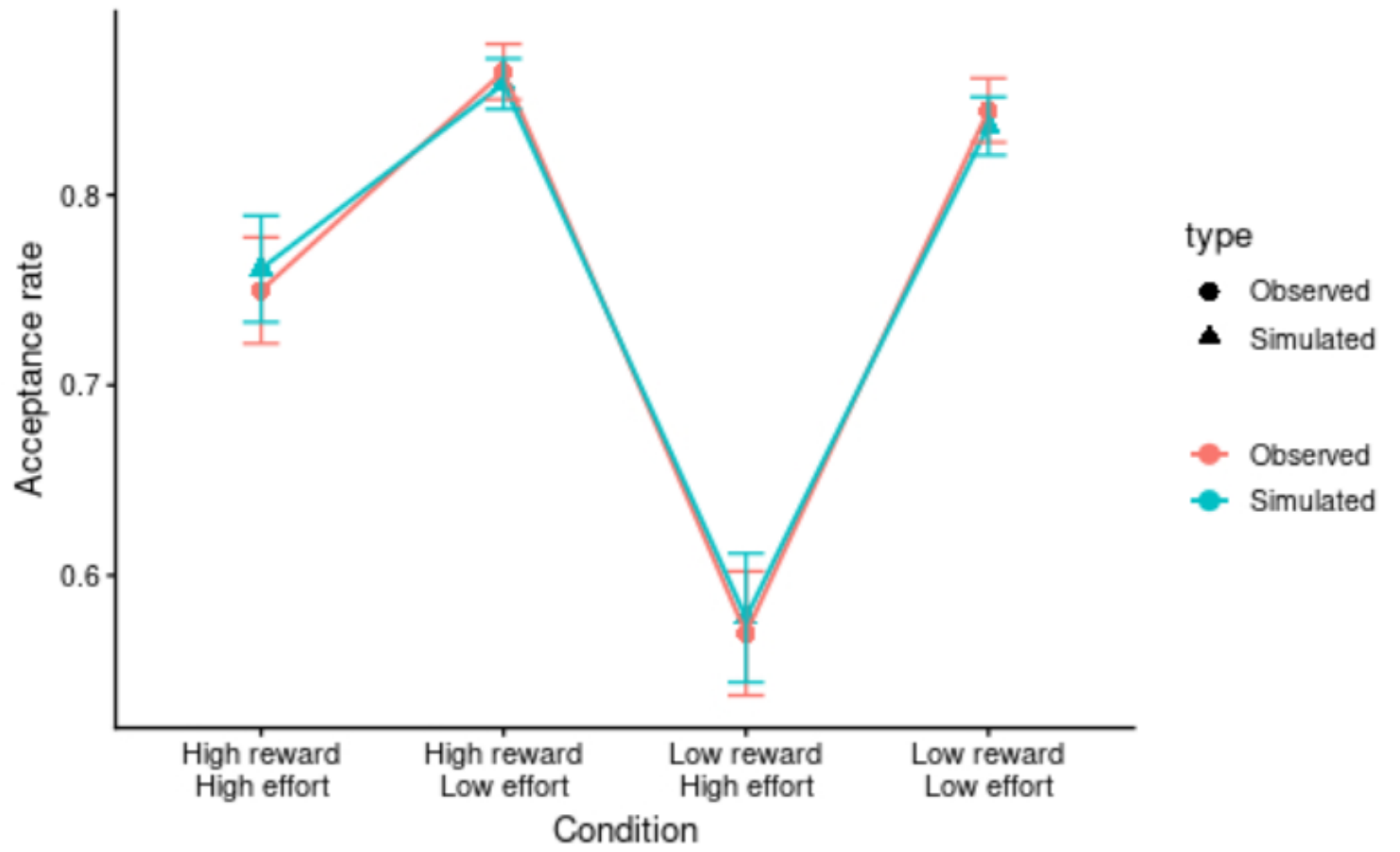

b. Effort x Controllability Interaction

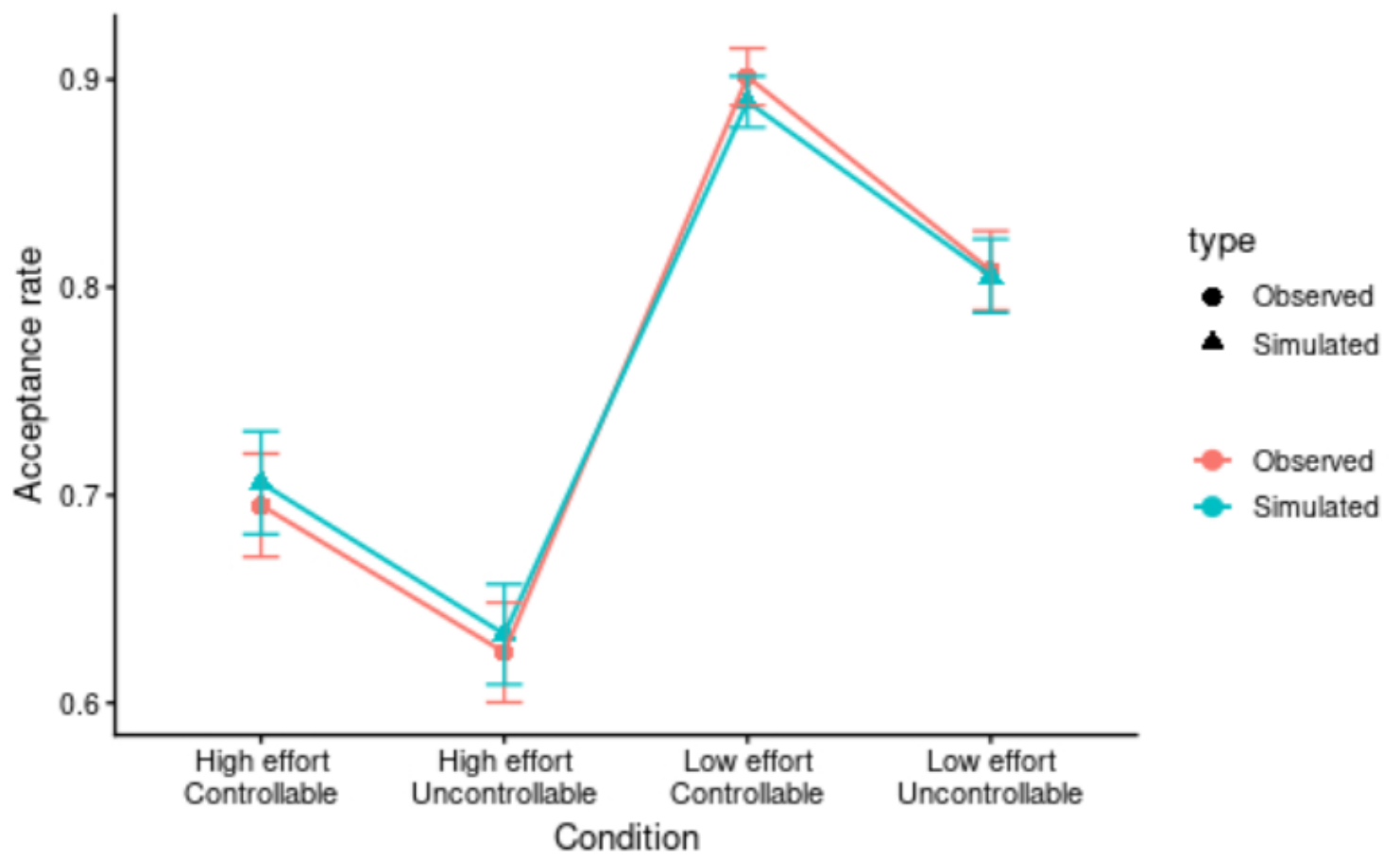

Figure S2.

Simulation-based predictive checks on the observed and simulated data on choice RT quantiles

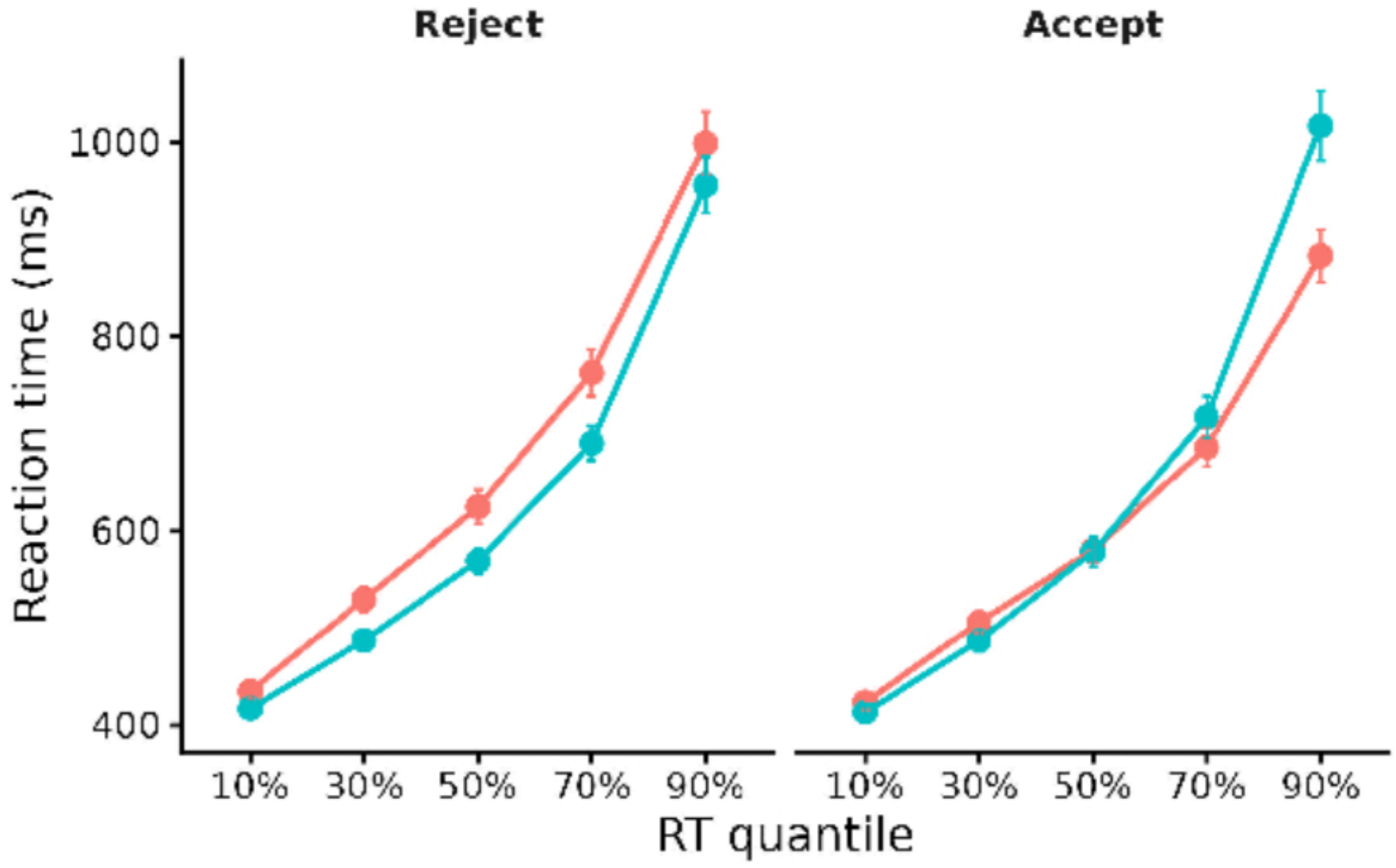

Figure S3.

Parameter recovery of the model estimation of the parameters.

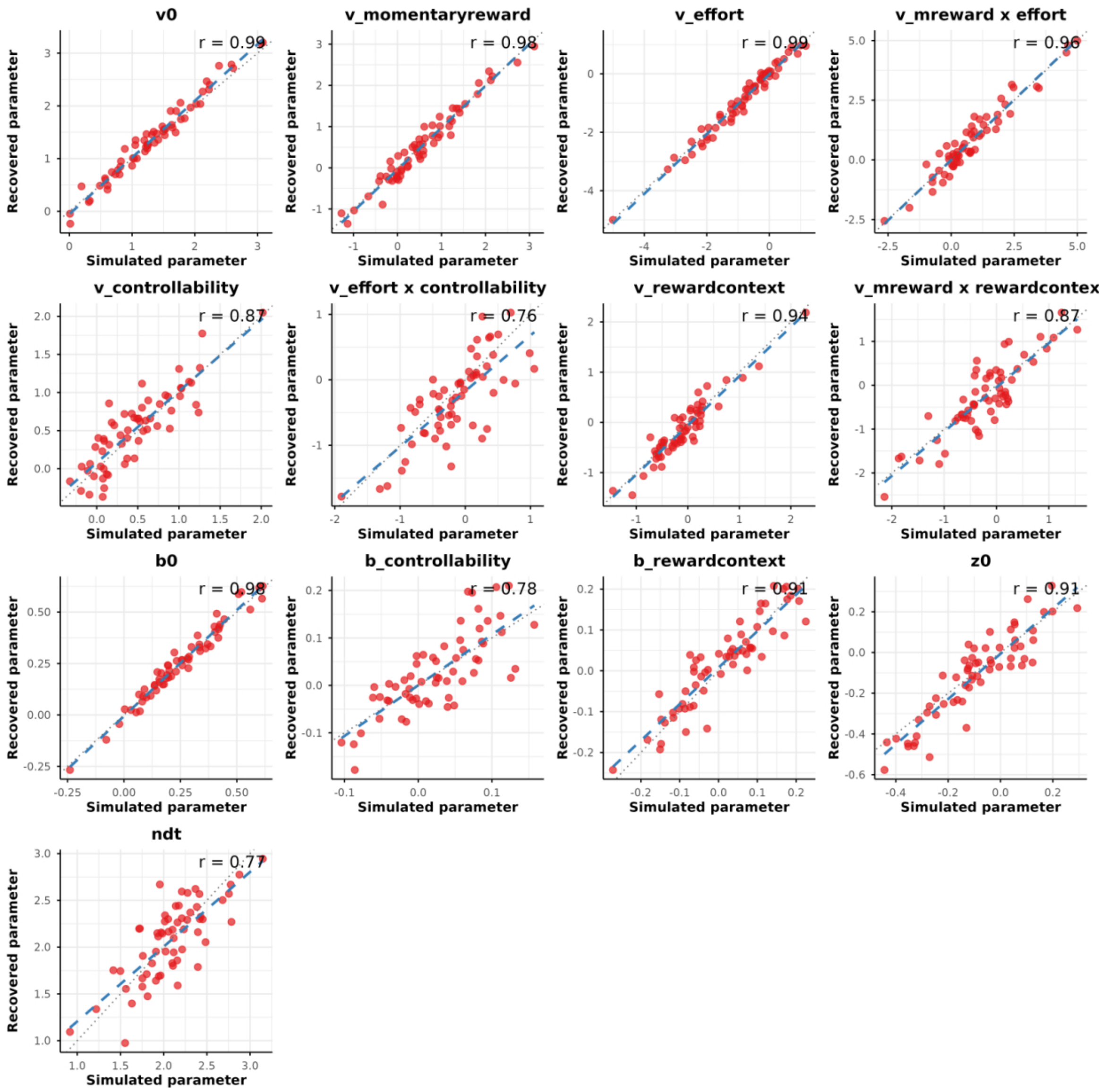

Figure S3. Parameter recovery results for the best-fitting DDM. Each panel shows one model parameter, and each red point represents one participant-level simulated and recovered parameter estimate. The blue dashed diagonal line indicates perfect parameter recovery, where simulated and recovered values are equal.
